# Community-based programs for youth with mental health conditions: a scoping review and practical implications

**DOI:** 10.3389/fpubh.2023.1241469

**Published:** 2023-11-02

**Authors:** Hila Tuaf, Hod Orkibi

**Affiliations:** Drama & Health Science Lab, Faculty of Social Welfare and Health Sciences, University of Haifa, Haifa, Israel

**Keywords:** adolescents, youth, mental health, recovery, community, leisure activities

## Abstract

**Background:**

Approximately 14% of all adolescents globally cope with mental health conditions. However, community-based psychosocial services for adolescents with mental health conditions are scarce and under-researched. Scant scholarly attention has been paid to leisure and/or social activities in community-based rehabilitation services for adolescents with mental health conditions.

**Objectives:**

To begin to fill this gap, we chose a bottom-up framework to probe the following questions: Which community-based programs for adolescents with mental health conditions exist worldwide? What common characteristics do they present? What is their range of services?

**Method:**

We systematically searched three leading academic databases, reference lists, and worldwide websites in English.

**Eligibility criteria:**

Programs with information in English that provide services in a community setting, service content that includes leisure and/or social activities, cater to users aged 10–18, and content explicitly targets adolescents with mental health conditions.

**Results:**

Twenty-seven psychosocial programs that provide leisure and/or social activities and encourage the promotion of adolescent mental health in the community were identified. We mapped and categorized the programs into three groups: integrated recovery, leisure recovery, and advocacy recovery.

**Conclusion:**

Practical implications for implementation are suggested based on the findings. Specifically, service providers should attend to the psychological needs of adolescents by prioritizing peer interaction and offering suitable social and leisure activities. These activities can also boost adolescent participation in community-based rehabilitation programs and address the treatment gap. Comprehensive studies and uniform terminology in the field are needed.

## Introduction

One in seven (14%) of all 10 to 19 year-olds have mental health conditions (MHC). This accounts for 13% of global morbidity among adolescents (1), and its prevalence is expected to increase (2). Scant attention has been paid to services that provide leisure and/or social activities in community-based rehabilitation services for adolescents with MHC. According to the World Health Organization (3), community-based rehabilitation (CBR) programs aim to

“promote and protect the rights of people with mental health problems, support their recovery and facilitate their participation and inclusion in their families and communities. CBR also contributes to the prevention of mental health problems and promotes mental health for all community members.” (p. 5)

However, our initial exploration indicated that many CBR programs for adolescents with MHC are presented online and not in academic publications, which curtails knowledge transfer on this topic. Consequently, we elected to implement the scoping review method which is commonly used to identify, map, and characterize the available data (4). The overall objective of this study was to provide stakeholders and policymakers with information on available CBR programs worldwide for adolescents with MHC. To the best of our knowledge, this study is the first to address programs that offer social and leisure activities in the community, which are of crucial importance for adolescents with MHC who may be at even greater risk of experiencing social challenges than age-appropriate challenges (5, 6).

### Adolescents with mental health conditions

Mental health conditions are an umbrella term for a range of psychiatric diagnoses including emotional disorders (e.g., anxiety and depression) of which anxiety is the most common among adolescents; behavioral disorders (e.g., attention deficit hyperactivity disorder and conduct disorder); eating disorders (e.g., anorexia nervosa and bulimia nervosa); psychosis; suicide and self-harm, as well as risk-taking behaviors (i.e., substance use or sexual risk-taking). Beyond age-appropriate challenges (6), adolescents with MHC are particularly vulnerable to social exclusion, discrimination, and stigma which affect their readiness to seek help (1) and are the main cause of the treatment gap (7, 8). The results of a recent systematic review indicated that 92% of all adolescents with MHC view social factors (e.g., public stigma and embarrassment) as barriers to seeking help (9). The mental health literature differentiates between public stigma and self-stigma (10). Public stigma is characterized by harmful labeling, prejudice, stereotype, and discrimination of a group of people to segregate them from society, whereas self-stigma (or internalized stigma) is characterized by the internalization of the public stigma in a way that influences people’s self-perception (10). Given the above, it is critical for service providers to consider the specific needs of adolescents with MHC during the recovery process, and tailor services with sensitivity and professionalism to suit them.

### The recovery approach for adolescents

For many years, the Westernized mental health system was dominated by the perception of individuals with MHC as patients who should be hospitalized for extended durations to reduce their symptoms (11). However, in recent decades, the personal recovery approach has increasingly influenced the rehabilitation policy of mental health systems (12). This approach is based on the person-centered principle and focuses on the improvement of these individuals’ quality of life despite their symptoms, thorough integration into the community, and restoration of a sense of control, independence, choice, autonomy, meaning, responsibility, and hope (11, 12). Clinical rehabilitation refers to an objective definition evaluated by a professional, such as reducing symptoms, whereas personal recovery refers to individuals’ self-perception of their own subjective recovery process (13, 14). In practice, the personal recovery approach takes the form of adolescent-oriented services that consider their age-appropriate developmental needs for independence, self-efficacy, and self-determination (15–17). As consumers of services, adolescents are encouraged to have agency in their recovery process, engage actively in decision making, and express their opinions and needs about these services (15, 18, 19). Evaluation studies of adolescent-oriented programs show high user satisfaction, especially with teams described as sociable, respectful, understanding, and non-judgmental (20). One emerging attempt to promote adolescents’ mental health is the “One Stop Shop” service, described next.

### Integrated youth health care: “One Stop Shop”

Stakeholders in the last decade have acknowledged the need to develop adolescent-oriented services to reduce the treatment gap (21, 22). These prevention, treatment, and rehabilitation services are tailored to be adolescent-friendly, inviting, engaging, and responsive to their needs (15, 23). Adolescent-oriented reforms have been launched in several countries, including Australia, the United Kingdom, Canada, and the Republic of Ireland (24, 25). These reforms aim to develop integrative holistic services in a single center that provides multidisciplinary care of physical health, mental health, and social services dubbed “integrated youth health care: one stop shop” (20). Studies show that the integrative service approach increases accessibility and attracts more adolescents to the service (26, 27). Most programs, however, focus on primary care (e.g., referrals to mental health professionals) and do not provide the much-needed afterschool leisure and social activities (20) that are reviewed here.

To date, publications on services for adolescents with MHC have focused mainly on the need for therapeutic interventions (28), and for integrated services (17, 19, 20, 23, 29, 30). In contrast, to the best of our knowledge, only two publications explicitly offer guidelines for youth mental health services in the community (15, 31). Moreover, many programs for adolescents with MHC are presented online and not in academic publications, which limits knowledge transfer on this topic.

## The present study

Despite the World Health Organization’s recommendation to increase CBR services, little scholarly attention has been paid to such programs. As part of a larger research project, the specific goal of the present study was to provide stakeholders and policymakers with information on CBR programs worldwide. To begin to fill this gap in the literature, we chose a bottom-up scoping framework to probe the following questions: Which community-based programs for adolescents with MHC exist worldwide? What common characteristics do they present? What is their range of services? Accordingly, this scoping review did *not* aim to assess program effectiveness or quality, but rather to identify, map, and characterize programs for adolescents with MHC that provide leisure and/or social activities in the community. Overall, this scoping review innovates by providing a comprehensive and detailed overview of community-based programs for adolescents with MHC that is likely to be of value to policymakers, stakeholders, and professionals.

## Method

**Figure 1:**
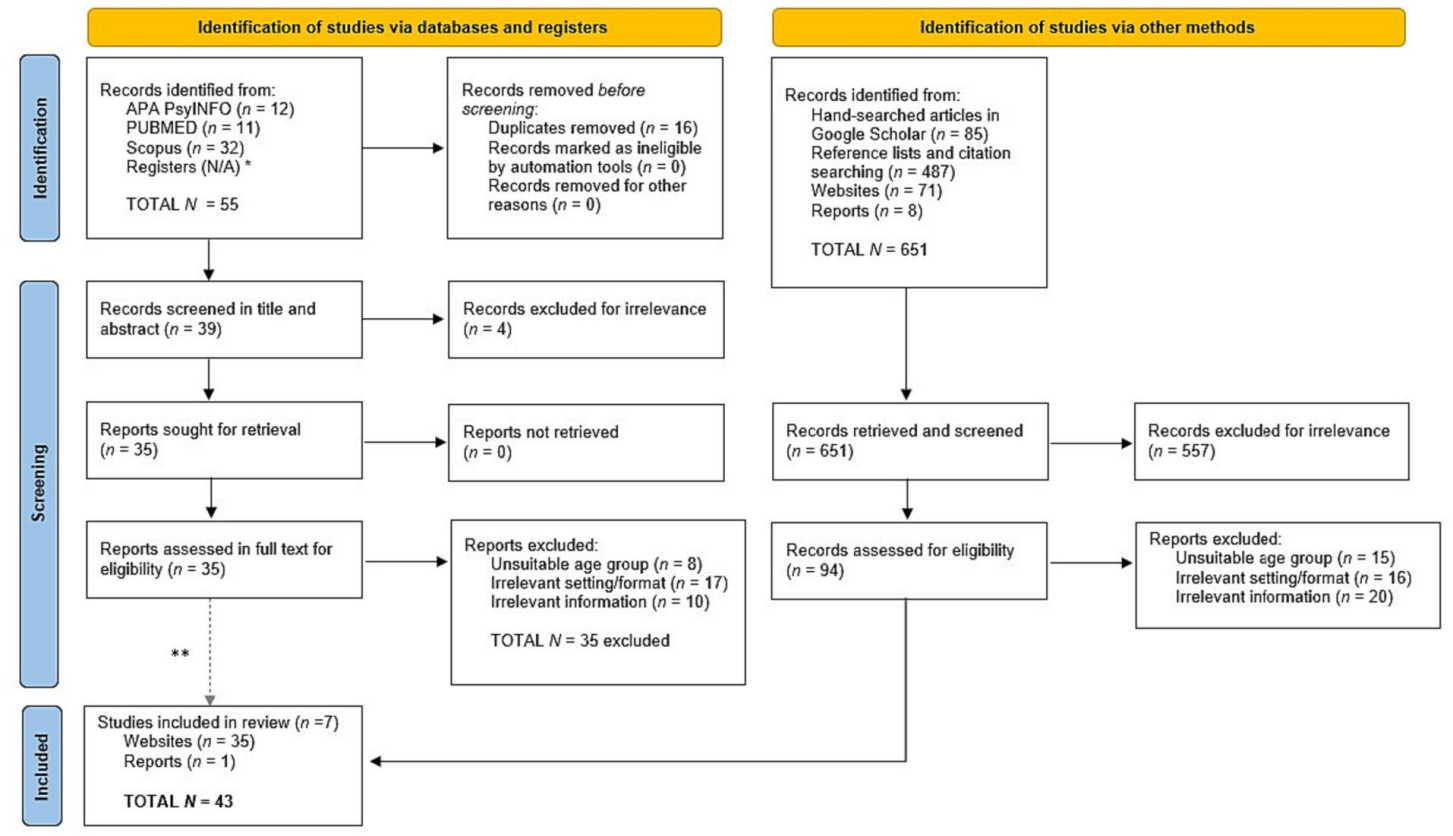
Prisma flow diagram. PRISMA 2020 flow diagram for new systematic reviews which included searches of databases, registers, and other sources. *Registers are N/A because we did not search registers of clinical trials since we excluded programs that only provided therapy interventions and services. **Note that none of the 35 reports assessed in full text for eligibility was included.

This scoping review adheres to the guidelines of the PRISMA-ScR Checklist for scoping reviews (4) wherever feasible (see Supplementary Appendix S1). Figure 1 presents the PRISMA flow diagram that shows the flow of results through the review process, from retrieval through screening and assessment of eligibility, to inclusion. Both authors developed the search strategy (see examples of the search strategy in Supplementary Appendix S2), and the first author (HT) conducted the search with the assistance of a specialist librarian. From 2018 to 2022, we first searched for programs in three major academic databases (Scopus, PsycInfo, PubMed) using the following search terms in the title and abstract: adolescents OR teenagers OR youth, AND “mental health condition” OR “mental illness” OR “mental disorder,” AND psychosocial OR rehabilitation OR recovery, AND program OR service, AND evaluation OR assessment, AND community OR leisure. There was no time limit on the search. As can be seen in the PRISMA flow diagram (Figure 1), on the left side (identification of studies via databases and registers), the database search retrieved 55 records, 16 duplicates were removed before screening, 39 records were screened for the title and abstract, and four were excluded due to irrelevance to the topic. The remaining 35 records were screened in full text to identify any names and information on CBR programs. However, all these 35 records were excluded for three reasons: unsuitable age group (*n* = 8, e.g., age younger than 10 and older than 18), irrelevant setting/format (*n* = 17, e.g., juvenile prison), and irrelevant information for the research questions (*n* = 10). However, some of these records led to reference list and citation searches, which yielded one program.

As can be seen in Figure 1, on the right side (identification of studies via other methods), we also conducted a hand search in Google Scholar using the same search strategy. This search yielded 85 records. At the same time, we searched these records’ reference lists and citations, which resulted in 487 records. When a program’s name was identified, we searched Google Scholar for more studies about it. However, the results were scanty, since in most cases there was little information on the characteristics of the program particularly with regard to leisure and social activities. Therefore, we searched the Internet for the program’s official website, which was sometimes also short or vague, which thus obligated us to search for more information on the Internet. We also expanded the Internet search for gray literature which yielded 71 websites and eight reports. This search led to 651 records that were retrieved and screened. Of these, 557 were excluded due to irrelevance to the topic. A total of 94 records were assessed in full text for eligibility, out of which 51 were excluded for three reasons: unsuitable age group (*n* = 15), irrelevant setting/format (*n* = 16), and irrelevant information (*n* = 20). Thus, in total, 43 records were included in this scoping review.

We provide a narrative description of the programs in the Findings section because the description of program characteristics varied depending on the information that was available from various sources. The basic information is presented in Tables 1–3. To provide as broad an overview as possible, we included all the programs that met the criteria even when their description was scarce. No study protocol was registered for this scoping review. During the revision process, we conducted an updated search in databases on August 15, 2023, which resulted in no inclusions.

**Table 1:** Leisure recovery programs.

#	Country	Program name	Website	Stated target population	Research report
1	Australia	Mind	https://www.mindaustralia.org.au/	Ages 16–46, with no specification of particular MHC	Yes
2	Australia	Young People’s Outreach Program	https://www.flourishaustralia.org.au/about/major-projects-funders	Ages 17–25, with no specification of particular MHC	Yes
3	Australia	Youth Community Living Support Service	https://www.wellways.org/our-services/youth-community-living-support-service	Ages 16–24, with a partial specification of MHC	Not mentioned
4	United States	Transition Age Youth Living Realized Dreams	http://www.taylrd.org/	Ages 16–25, with no specification of a particular MHC	Not mentioned
5	United States	The Drop Model	https://www.youthera.org/drop-in-centers	Youth, with no specification of a particular MHC	Yes
6	Canada	LOFT Transitional Age Youth (TAY)	https://www.loftcs.org/	Ages 12–26, with emotional and behavioral disorders and risk-taking behaviors	Yes
7	United Kingdom	Reeltime Music	https://www.reeltimemusic.net/	Ages 12–18, with no specification of a particular MHC	Not mentioned
8	United Kingdom	Aye Mind	http://ayemind.com/	Youth, with no specification of a particular MHC	Yes
9	United Kingdom	The Junction	http://the-junction.org/	Ages 12–21 with emotional disorders and risk-taking behaviors	Not mentioned
10	Finland	YEESI	https://yeesi.fi/	Ages 13–29	Yes
11	Israel	Amitim for Youth	https://www.amitim.org.il/blank-10	Ages 12–18 with emotional, behavioral, and eating disorders	Yes

Ages are reported numerically when specified by the program. The term “youth” is used in programs designed for adolescents and young adults. The term “partial specification of MHC” consists of emotional disorders, behavioral disorders, eating disorders, psychosis, suicide, self-harm, and risk-taking behaviors.

**Table 2:** Integrated recovery programs.

#	Country	Program name	Website	Stated target population	Research report
1	Australia	Headspace	https://headspace.org.au/	Ages 12–25, with a partial specification of MHC	Yes
2	New Zealand	The Youth One Stop Shop	http://www.yoss.org.nz/home.html	Ages 10–25 with emotional disorders and risk-taking behaviors	Yes
3	United States	Supporting Positive Opportunities with Teens	http://thespot.wustl.edu/	Ages 13–24, with emotional disorders and risk-taking behaviors	Not mentioned
4	Canada	Youth Wellness Hubs Ontario	https://youthhubs.ca/en/	Ages 12–25, with emotional and behavioral disorders and risk-taking behaviors	Yes
5	Canada	ACCESS Open Minds	http://accessopenminds.ca/	Ages 11–25, with no specification of a particular MHC	Yes
6	Canada	Foundry (BounceBack)	https://foundrybc.ca/	Ages 12–24 with emotional and behavioral disorders and risk-taking behaviors	Yes
7	France	Association Nationale Maisons des Adolescents	https://anmda.fr/	Ages 11–25, with no specification of a particular MHC	Yes

Ages are reported numerically when specified by the program. The term “youth” is used in programs designed for adolescents and young adults. The term “partial specification of MHC” consists of emotional disorders, behavioral disorders, eating disorders, psychosis, suicide, self-harm, and risk-taking behaviors.

**Table 3:** Advocacy recovery programs.

#	Country	Program name	Website	Stated target population	Research report
1	United States	Youth MOVE National	https://youthmovenational.org/	Youth, with no specification of particular MHC	Yes
2	Canada	Jack.org	https://jack.org/Home	Youth with emotional disorders, suicide, and self-harm	Yes
3	United Kingdom	YoungMinds	https://youngminds.org.uk/	Ages 14–25, with a partial specification of MHC	Yes
4	United Kingdom	Time To Change	https://www.time-to-change.org.uk/	Youth, with no specification of a particular MHC	Yes
5	The Republic of Ireland	Jigsaw	https://www.jigsaw.ie/	Ages 12–25, with a partial specification of MHC, except psychosis	Yes
6	Netherlands	The Dutch National Youth Council (NJR)	https://www.njr.nl/en/	Ages 12–30	Not mentioned
7	Israel	Headspace	https://headspace.org.il/	Ages 12–25 with emotional, behavioral, and eating disorders, and risk-taking behaviors	Yes
8	India	It’s Ok To Talk	http://itsoktotalk.in/	Youth, with a partial specification of MHC	Yes
9	Singapore	CHAT-Community Health Assessment Team	https://www.chat.mentalhealth.sg/	Ages 16–30, with a partial specification of MHC	Yes

Ages are reported numerically when specified by the program. The term “youth” is used in programs designed for adolescents and young adults. The term “partial specification of MHC” consists of emotional disorders, behavioral disorders, eating disorders, psychosis, suicide, self-harm, and risk-taking behaviors.

### Inclusion criteria

The inclusion criteria for programs were: (a) programs with information in English from publications or websites that have an English version, (b) caters to users aged 10–18, (c) provides services in a community setting, (d) content explicitly targets adolescents with MHC and/or related psychosocial difficulties, and (e) services must include leisure and/or social activities. We established a broad definition of leisure and social activities to encompass all types of activities in the community, including those that adolescents engage in during their free time for recreation, hobbies, or socializing with peers that are thought to promote personal recovery in adolescents (12, 15). This includes arts, sports, peer-group activities/gatherings, advocacy, community events, watching movies, etc. Note that in line with the World Health Organization’s definition of CBR (3), we excluded programs delivered at schools, hospitals, and clinics, because they are not considered to be community-based (30). Note that the concept of “community” in CBR includes not only the physical neighborhood but also the social networks and support structures that surround individuals. Thus, for example, while schools and hospitals are essential components of a community’s infrastructure, they are typically not classified as CBR because they are institutional settings that offer specialized services in education or health, with a primary pedagogical or clinical orientation, respectively. Although these institutions may provide psychosocial support services, their core mission is different from the holistic approach of CBR, which has a de-centralized holistic approach to rehabilitation service-delivery aiming to promote the overall well-being, social inclusion, and active participation of individuals with MHC in their communities, as opposed to participation in specialized institutions or facilities (3, 32). On this basis, this scoping review sought programs that are delivered during the leisure time of youth with MHC. Therefore, our focus was on services that promote social integration and leisure activities in the community, explicitly after school hours. We also excluded programs that only provided therapy services or therapeutic interventions since they do not provide leisure and/or social activities in the community.

### Data extraction

All 27 programs that were identified were examined in-depth by the first author (HT). She systematically extracted data to a spreadsheet with the following information from the program websites, when available: country, program name, target population, users’ age range, stated program aims, program’s physical location, funding sources, years of operation, services and activities offered, and studies, if any, conducted on the program. The websites of the programs that met the criteria were vetted for authenticity (e.g., formal program websites) by both authors to ensure that they met the eligibility criteria. Thirteen cases where there was uncertainty were discussed by the two researchers until a mutual decision could be reached.

### Data analysis

The data analysis drew on the thematic analysis procedure that is often used in psychology to identify meaningful patterns in textual data (33). In step 1, the first author (HT) familiarized herself with the entire dataset through an iterative process of reading through all the information available on each program. In step 2, HT systematically conducted a comprehensive identification of each programs’ leisure and/or social activities, beyond psychosocial support. This led to step 3, where HT generated initial categories. In step 4, to enhance the rigor of the analysis, a double review process was implemented where the second author (HO) independently reviewed the initial categorizations of the first author. Any areas of uncertainty or potential discrepancies in the categorization were addressed through collaborative discussions between the two authors. This dialog aimed to achieve a consensus-based decision on the assignment of each program to its respective category. Subsequently, in step 5, the authors refined the three program types as leisure recovery programs, integrated recovery programs, and advocacy recovery programs (see Findings section). Finally, in step 6, a descriptive report of programs was written with findings presented in narrative and three tables.

## Findings

A total of 43 items were included in this scoping review: 7 studies, 35 websites, and 1 report. Twenty-seven community-based programs worldwide were identified and vetted independently by both authors (Tables 1–3). Most of these programs (26/27 programs) specified their target population as “adolescents” and/or “young adults” who have MHC, however without explicitly specifying the consumers’ particular psychiatric conditions. The use of this broad terminology seemed purposeful, and designed to avoid labeling and stigma to reach out to a wider population. A few programs (8/27) are offered as part of a broader service, foundation, organization, movement, or charity that provides psychosocial support to adolescents with MHC. Most of these programs have not been reviewed in academic publications, and when they are, the program’s leisure, social and community activities are not specified. After identifying the programs and their main components, we mapped and characterized them into three groups, to address the research questions of what range of services they offer: leisure recovery programs (Table 1), integrated recovery programs (Table 2), and advocacy recovery programs (Table 3). The tables list each program’s country of origin, website, stated target population, information on MHC if stated, and whether the program has been researched.

### Leisure recovery programs

Leisure recovery programs offer adolescents social and leisure activities in the community, given their age-appropriate need for self-definition and socialization with peers. Leisure recovery programs generally do not provide physical health services, as can be seen in Table 1.

The Australian *Mind* program was founded over 40 years ago for adolescents and adults aged 16–46. *Mind* offers creative leisure activities, social activities, skill acquisition, and peer support groups where users with MHC provide social support to other users with MHC (see Table 1, Item 1). Another program in Australia is the *Young People’s Outreach Program (YPOP)*, where peer support youth workers mentor and support users aged 17–25 in life skills such as housing, employment, and sustaining healthy activities and relationships (see Table 1, Item 2). *YPOP* is part of the *Flourish Australia* community services that have operated for over 60 years and are funded by the Australian government. *Flourish Australia,* for example, organized a “graduation-style formal evening” for former and present users aged 16–24 with MHC who could not participate in the past due to MHC and other issues (34). Given the success of the *YPOP*, the *Youth Community Living Support Service* (YCLSS) was established in 2016 in Australia, funded by the New South Wales (NSW) government (35). The team encourages its users to participate in community activities, pursue education and employment. The team offers psychosocial support and case management for adolescents and young adults aged 16–24, and takes an outreach approach by providing early interventions to the users at their location (see Table 1, Item 3). *Transition Age Youth Living Realized Dreams (TAYLRD)* in the US operates 16 drop-in centers in Kentucky, for users aged 16–25. These drop-in centers designed together with young adults offer safe, convenient, and enjoyable spaces where consumers are supplied with free snacks, a kitchen, washers and dryers, and various leisure time activities including an art room, media room, 3D gaming and movies, a computer lab, and a pool table. A range of services are offered, including life skills, goal setting, peer support, case management, psychiatric care, therapy, academic support, court legal support, employment and education services (see Table 1, Item 4). In Oregon, *The Drop Model* program was established in 2017 and operates five youth clubs (“drop-in” centers), which hold meetings, social activities (games, movies), work towards the development of leadership skills, peer support and provide educational support (help with homework) inspired by the Headspace model (see Table 1, Item 5). This program is part of the *Youth ERA* organization that also provides training to adolescents and young adults to become peer supporters (also termed “peer workforce”) in 39 states across the United States (36).

The *LOFT Transitional Age Youth (TAY)* program in Canada is intended for adolescents and young adults aged 14–26 with MHC (and/or complex conditions such as substance use, physical health issues). The staff aims to increase personal recovery by providing peer support groups, life skills and social groups, education, employment support and three youth wellness hubs (“drop-ins”) in Toronto, and case management through an outreach approach (meeting users at a place of their choosing). *TAY* is offered as part of the *LOFT* (Leap of Faith Together) mental health service and is a charity that was established in 1953, which is funded by the Province of Ontario and donations (see Table 1, Item 6).

The *Reeltime Music* program has been operating since 1997 in the United Kingdom as a musical-social project where adolescents with MHC (aged 12–18) raise awareness of mental health issues by engaging in rehearsals, recordings, and performances at festivals. These adolescents run campaigns in the media and interview people on their opinions about mental health (see Table 1, Item 7). Another program in the UK is *Aye Mind*, which was founded in 2013. The program includes workshops where making GIF animations serves as a creative outlet for adolescents to express their attitudes and feelings about mental health and well-being. They publish their creative output in the media as part of a campaign to reduce stigma related to MHC (see Table 1, Item 8). *The Junction* is another program in the UK that enables users with MHC (aged 12–21) to get involved in creative projects such as poetry, painting, collage, creative writing, etc. The program also offers personal support services and counseling (see Table 1, Item 9).

The *YEESI* program was established in 2011 in Finland by the Finnish Ministries of Health and Welfare in collaboration with adolescents who have MHC. *YEESI* engages users aged 13–29 in community-based volunteer activities where they can also serve as members of the organization at annual board meetings. The program operates youth centers called *Yeesi points* in the community and schools. These locations enable adolescents to connect, support each other, initiate social activities, and receive support such as educational assistance (see Table 1, Item 10).

In Israel, the *Amitim for Youth* program was established in 2018 by the Ministry of Health, the Ministry of Education, and the Special Projects Fund of the National Insurance Institute. This program has been implemented in six regions by the Israel Association of Community Centers and is intended for adolescents with MHC aged 12–18. The *Amitim for Youth* team facilitates social integration in the community with an outreach approach that involves encounters with end-users (adolescents and their parents) in the community and their homes. They provide guidance and support from coordinators and volunteers, through individual sessions, social activities for adolescents with and without MHC, creative leisure activities, and social skill acquisition groups. The program team cooperates with other caregivers to share information and maintain continuity of care, such as schools, clinics and social services, and also initiates advocacy activities for adolescents in the community to reduce stigma, for example by screening movies on mental health followed by a discussion (34, see Table 1, Item 11).

### Integrated recovery programs

Integrated recovery programs provide holistic service to users that include leisure and/or social activities along with physical health and mental health services, as shown in Table 2.

In Australia, the *Headspace* National Youth Mental Health Foundation was established in 2006 by the Australian Government Department of Health. *Headspace* operates in more than 100 centers across Australia in cities, towns, and villages. The centers provide an integrative “one-stop-shop” for adolescents and young adults aged 12–25. The team cooperates with various organizations for optimal support (schools and social services), and hosts concerts, skating and gaming events in the community (26). The program also nurtures young opinion leaders, including volunteers and graduates of the *Headspace* center who provide feedback to the program’s team on their services. In addition, they provide support to young people, and offer advocacy activities for stigma reduction in centers, communities and social media (see Table 2, Item 1). Evaluation studies conducted on *Headspace* in Australia have shown that adolescents and family members expressed high satisfaction with the service (17, 26, 37), and that nearly half of the consumers described feeling less mental distress at the end of treatment (26). The Australian *Headspace* model has been implemented in other countries.

The *Youth One Stop Shop (YOSS)* has been operating since 1994 in New Zealand. This integrative service is funded by the Ministry of Health and private donations. The service is provided in 14 centers, in a youth environment that includes a common seating area, a pool table, music and arts activities, all designed to create a sense of belonging to the community. In addition, the service offers life skills programs for users aged 10–14. YOSS provides psychosocial and medical care to adolescents and young adults aged 10–25 and their families (see Table 2, Item 2).

Another integrative service in the United States is the *Supporting Positive Opportunities with Teens (SPOT)*, established in 2008 in Missouri. This integrative service operates a youth center (the SPOT youth center) for adolescents and young adults aged 13–24, where a variety of essential facilities are provided (e.g., laundry, shower, kitchen, and computers). In addition, the members can participate in structured activities to impart social skills and reduce stress, including arts, games, cooking, watching movies and lectures. The SPOT also provides medical and social services, support calls, counseling, and referrals to various mental health professionals (see Table 2, Item 3).

In Canada, the *Youth Wellness Hubs Ontario (YWHO)* was founded in 2017 in Ontario. *YWHO* is an integrative (psychosocial, educational, medical, training, employment, and housing) government service in four centers that addresses the treatment gaps in the mental health system for users aged 12–25. The team offers peer support as well as leisure and social activities such as outdoor activities, arts and crafts, drama, music, board games, bowling, cooking, athletics, etc. The service has been evaluated through surveys, and the teams participate in the decision-making process in local and district committees (see Table 2, Item 4). A study conducted on records of 1,520 users aged 17–25 found that young people made most referrals themselves. Users rated their service satisfaction as 3.37 and the service quality as 3.72 out of 5 (38). The Canadian service *ACCESS Open Minds* was established in 2014 to turn mental health clinics into integrative service centers providing medical and psychosocial care for users aged 11–25. It operates 14 centers, some of which have youth clubs with leisure activities (see Table 2, Item 5). Another integrative service in Canada is *Foundry*, which was established in 2015 and provides medical and psychosocial care to users aged 12–24 and their families. The service is provided in 11 centers in eight communities in British Columbia. The centers offer wellness programs such as peer support groups, meditation and outdoor sports. Another program in the service is the *BounceBack* program, which provides tools for improving mental health for users aged 15 and above who are coping with depression, stress, or anxiety, and is led by a personal trainer, or through a self-help booklet and videos on their website. In addition, the *Foundry* website provides several ways to obtain support, including via phone, chat, links to apps, games and guides for mental help, as well as support for friends and family (see Table 2, Item 6).

In France, the *Association Nationale des Maisons des Adolescents* was established in 2004. This integrative service operates 104 centers that offer psychosocial and medical care for users aged 11–25. The centers provide a variety of leisure activities including arts and painting, radio and musical performances, sports activities, cooking, gardening, dance, fashion, hair design, music, comedy shows, movies and literature courses. In addition, the centers provide an environment suitable for youth with a seating area, garden, cafe, and library (see Table 2, Item 7).

### Advocacy recovery programs

Advocacy recovery programs aim to promote awareness of mental health issues by encouraging youth with MHC to engage in campaigns for stigma reduction that empower those involved. Advocacy recovery programs generally do not offer physical or mental health services, but they do include leisure and/or social activities (e.g., community activities), as shown in Table 3.

In the United States, the *Youth MOVE National* program was established in 2007. *MOVE* stands for “Motivating Others through Voices of Experience.” The program’s main goal is to promote the participants’ rights through community involvement in 60 branches across 35 states (see Table 3, Item 1).

The Canadian charity *Jack.org* trains young leaders to promote positive mental health through three programs: *Jack Talks, Jack Chapters,* and *Jack Summits*. Each program is partnered with sponsors. In *Jack Talks*, young trained speakers share their personal stories and educate young audiences to inspire and equip them to care for their own and their friends’ mental health. In *Jack Chapters*, groups of young trained advocates (mostly high school and college students) learn to break down barriers to positive mental health in youth activities in their communities. In *Jack Summits*, youth-led conferences throughout Canada strengthen leadership and advocacy skills to implement strategies for change. In 2019, *Jack.org* launched the *BeThere* website that teaches “five golden rules” to support individuals experiencing mental health difficulties. The charity also hosts large social fundraising events such as *Jack Riders* (group bike-riding) and *Brainfreeze* (group immersion in icy water). *Jack.org* was founded in 2010 and is supported by the Government of Canada’s Emergency Community Support Fund and the Community Foundation of Nova Scotia, Toronto Foundation, Edmonton Community Foundation and Community Foundation of Mississauga. The teams are mostly composed of young adults who take a young and light-hearted youth-friendly attitude: they even post their dogs on the website (on the staff page) in positions such as “cuddle coordinator” and “hugger-in-residence” (see Table 3, Item 2).

Another program to empower youth is the *YoungMinds* movement in the United Kingdom, which offers four main programs for users aged 14–25: *Youth Panel, YoungMinds Activist, YoungMinds Blogger* and *YoungMinds App Tester*. In *Youth Panel,* users advise, engage with and co-influence (with the senior management team) operations, campaigns, resources and fundraising; in *YoungMinds activist*, users with MHC or those experienced with helping a person with MHC, acquire campaigning, facilitating, and presenting skills in training, where they meet and connect with their peers. In *YoungMinds Blogger*, users share their personal stories and advice on the *YoungMinds* website. In *YoungMinds App Tester*, users review the development of the *YoungMinds* app, contribute to content, develop campaigns and influence government policies. *YoungMinds* was founded in the UK in 1993 as a charity to enable young adults to voice their views and raise mental health awareness. The *YoungMinds* team gives users the opportunity to engage in voluntary or paid roles in the movement’s offices, events, and campaigns. The team also encourages engagement in various activities, such as sporting events, activism, training, volunteering, blogging, participating in campaigns, conferences, and groups on social media. The movement also has a phone support line for parents of struggling adolescents (see Table 3, Item 3). Another social movement called *Time To Change* in the UK has been operating in community centers and schools since 2007. It enables adolescents with MHC to share their personal stories on the movement’s website and through campaigns on social media. The movement’s goal is to raise awareness of mental health, while reducing stigma and discrimination in the community (see Table 3, Item 4).

The *Jigsaw* program was established in 2006 (called *Headstrong* until 2016) in the Republic of Ireland. The program operates in 13 centers that provide psychosocial services for users aged 12–25. The program has a *Youth Advisory Panel* comprised of volunteers aged 16–25 who discuss issues such as decisions, leadership, the quality of the service and customizing it to the consumers. They also participate in various advocacy activities in the centers and the community (see Table 3, Item 5). An evaluation study conducted on the *Jigsaw* program with 2,420 participants (aged 12–25) who completed the Clinical Outcomes in Routine Evaluation questionnaires (CORE-10 and YP-CORE) reported a significant decrease in mental distress (27). In a study conducted in 12 *Jigsaw* centers, 510 parents of users (aged 12–17) reported high parental satisfaction with the service and positive outcomes for their children (39).

In the Netherlands, the *Dutch National Youth Council (NJR)* organization was established in 2001. Users aged 12–30 participate in an active political way to reduce stigma through the media by sharing their experiences on videos, and by writing reports that reflect their needs from the community and schools. The participants have presented their first document describing their main barriers to engaging in society at two European Union conferences (see Table 3, Item 6). The Australian *Headspace* model (described above) inspired the opening of a *Headspace* program in Israel. The first center in Israel was established in 2015 by the *Enosh* association in the city of Bat-Yam, and the second in 2019 in Jerusalem. The program is funded by the Special Projects Fund of the National Insurance Institute, local authorities, and private funds. The program team provides activities and special projects for adolescents and young adults (aged 12–25) including the *Headspace* ambassadors project where high school students (who are studying psychology) interact with the headspace team to raise awareness of mental health through advocacy, and collaboration with the community by training mental health professionals and educators. The team also provides social skill acquisition groups, short-term therapy, and a mentoring project where soldiers prepare adolescents with MHC for military service (see Table 3, Item 7).

*It’s Ok To Talk* was established in India and operated from 2016 to 2018, as part of a research project called PRIDE, in collaboration with Harvard University which was funded by the United Kingdom Wellcome Trust. As part of the program, adolescents and young adults participated in community activities (events and workshops) and created social media campaigns to raise mental health awareness. The program produced a website that enables youth to conduct a dialog on mental health, share personal stories, express their thoughts and emotions through arts and drawings, and ask and receive help (see Table 3, Item 8). The PRIDE research project also developed tools for self-help and learning about mental health, such as comic books and applications (40).

Finally, in Singapore, the *Community Health Assessment Team (CHAT)* was established in 2009. The *CHAT* team initiates partnerships, projects and campaigns in the community and media to reduce stigma and raise awareness of the center and its services and mental health through theater, arts, exhibitions, and filmmaking. In addition, the team provides support to users aged 16–30, training workshops for peer support, information regarding mental health, coordinates care and refers members to additional services (see Table 3, Item 9). A study conducted on the *CHAT* database reported that 73.9% of referrals were made by the participants themselves. In addition, since 2014 *CHAT* has also been operating *The CHAT Ambassadors Program* comprised of volunteers aged 16–30. As of 2021, 55 ambassadors had led and initiated projects and campaigns to improve quality of service, advocacy and users’ participation by redesigning the service’s facilities such as a youth center, a website, advocacy activities, as well as forums for users to evaluate the service (41).

In sum, the purpose of this scoping review was to find information on CBR programs that provide leisure and/or social activities for adolescents with MHC. A total of 27 psychosocial community-based programs were identified. Their common characteristics consist of (a) a vague specification of the target population, (b) an online presence, (c) providing leisure and social activities for adolescents, (d) offering peer support, and (e) applying different forms of advocacy to raise awareness of mental health in the community. Many of these programs are (f) located in youth centers, and (g) have a welcoming and friendly atmosphere. These programs provide adolescents with opportunities to interact with their peers, acquire new skills, and enhance their well-being.

## Discussion

The overall objective of this study was to provide stakeholders and policymakers with information on available CBR programs worldwide for adolescents with MHC. we chose a bottom-up scoping framework to probe the following questions: Which community-based programs for adolescents with MHC exist worldwide? What common characteristics do they present? What is their range of services? Twenty-seven community-based programs worldwide were identified, whose goal is to promote adolescent mental health in the community. Most of these programs (26/27 programs) specified their target population to be “adolescents” and/or “young adults” who have MHC, without directly specifying their end-users’ psychiatric conditions. It is likely that the use of this broad terminology is a deliberate step to avoid labeling and stigma. Similarly, most programs allow self-referral, without psychiatric diagnosis to enable the inclusion of wider spectrum of users and the promotion of mental health among adolescents. Allowing self-referral coincides with the personal recovery approach, where adolescents are encouraged to have agency and engage actively in their recovery (15, 18, 19). The practical implications for the implementation of community-based adolescent-oriented programs for service providers and researchers are discussed below.

### Service characteristics

*Adolescent-friendly facilities* can increase adolescent engagement in programs (19) and are perceived as less stigmatized when they are located in the community rather than in a mental health institution (42). Studies have indicated that adolescents’ unwillingness to receive professional help may be related to their fear of being stigmatized by their peers (43–45). Fear of stigma was reported to be the most common barrier to seeking service by young participants (aged 12–25) in the *Headspace* program in Australia who were concerned with being perceived as “crazy or psychotic” (26). However, the results suggested that young people may feel more at home in a center that is informal, comfortable and an inviting place that holds interesting fun events, such as concerts where adolescents perform (26).

This study confirms that programs operate adolescent-oriented facilities that are characterized by a welcoming friendly style and atmosphere, and offer content aimed to provide a relaxed, appealing non-clinical physical space to enable these adolescents to feel safe and comfortable (26). Some programs are held in attractive youth centers (drop-in/hubs) equipped for leisure time activities and have essential facilities such as kitchen and computers. Service providers should thus consider operating adolescent-friendly youth centers that would increase engagement and decrease fear of stigma.

Studies show that *leisure activities* can promote empowerment, a quest for meaning and improved social relations with peers and the community (46), and that self-selected activities are essential because they can enhance engagement and subjective well-being in adolescents (47). Studies on adults also suggest that leisure activities contribute to the recovery process, strengthen their sense of belonging, autonomy, competence, meaning, and hope while reducing depression, stress, and boredom (48–50). Out of 27 programs, 11 primarily provided leisure activities. Service providers should thus include social and leisure activities in programs and take adolescents’ psychosocial needs for peer interaction and self-efficacy into consideration. Both needs can be provided through structured leisure group activities that involve social skills such as art making, the performing arts, music, sports, and workshops to impart various skills for enjoyment and provide relief from stress.

In terms of *online presence*, all the programs operate an active website and most also have a social media presence. Online presence, including an inviting and vibrant website that targets young adults as well as an active representation on social networks (e.g., Instagram, Facebook, Twitter) are crucial for program visibility. Some programs do not provide information about their services and do not specify the activities and community events offered. This suggests that transparency, clarity, and full details on the websites are essential and may boost adolescent engagement as well as help reach a wider audience such as non-clinical adolescents or young adults. Active representation of the programs in the media and on social networks may allow for direct communication with adolescents (without the mediation of referrals), through an interface available to them in their own language. Some programs use their online presence to encourage user empowerment by providing a venue for youth to express themselves, share personal stories, offer peer support and serve as a platform for public health stigma reduction campaigns. Service providers should maintain an inviting online presence including on social media. Transparency, clarity, and full details are key factors to ease access, engagement and direct communication with adolescents.

Out of 27 programs, eight offer *peer support* by individuals who are close in age to the adolescents and who have also experienced adversity or overcame MHC. These individuals typically draw on their own experiences to assist and guide young participants (51–53). In recent years, more programs have begun implementing peer support in their services (51, 53). Although there are few studies assessing the effectiveness of peer support in mental health services (51, 53), studies on adults have shown that peer support programs positively affect the recovery process by enhancing engagement and reducing hospitalization stays (54, 55). Therefore, peer support is a key resource when providing services for adolescents with MHC (51). However, youth peer support may also be a challenge, given the need for training, supervision, clear job definitions (51, 53, 55), their young age and workload (51). Service providers should thus include peer support in their services, with an emphasis on training and supervision of support staff to ensure a professional response.

### Advocacy, community involvement and collaborations

Out of 27 programs, 12 (see all programs in Table 3, programs 1 and 11 in Table 1, and program 1 in Table 2) call on adolescents to engage actively in advocacy activities to reduce stigma. These programs encourage adolescents to get involved in campaigns to reduce stigma through events in the community and the media. Programs can benefit from partnerships with various community services which may also attract additional or exclusive funding from various donors, associations, and partners. Service providers may also gain from engaging members of the broader community in programs (17, 37). Holding events in the community by and for adolescents may raise community awareness about mental health and stigma. Service providers should thus initiate collaborations with the broader community to enhance inclusion, advocacy and awareness.

### Research and evaluation

Most programs state online that they employ an internal research team that also involves their consumers in the ongoing evaluation of the service. However, empirical evidence on these programs is limited; despite our thorough searches, few research publications on the programs were found. In addition, studies on these programs should be carried out by external, disinterested evaluators and researchers to minimize potential research biases, and disseminate the results transparently. Finally, programs and researchers sometime use different terminology to describe their target population (e.g., youth with MHC/issues/difficulties, mental illness, serious mental disorder etc.), and varying definitions of the service. Using a unified terminology would facilitate continuity of care and collaborations between services, address the treatment gap, ease accessibility to services for users and their families, increase the robustness of the literature, raise awareness and help promote a non-stigmatic discussion in the community about mental health. More empirical evidence in the field, maintaining unified terminology is needed.

## Limitations

This scoping review by no means provides an exhaustive account of all programs since it only includes programs with information in English from publications or websites that have an English version. Two programs in non-English languages (7 in Table 2 and 10 in Table 1) were mentioned in publications and examined using Google Translate. Further, given the scant empirical data on the topic, the data drew extensively on internet websites. It should be noted that except for India, all the programs are from developed and relatively high-resource countries. Another challenge was the difficulty locating programs for adolescents with MHC, because the related terms (e.g., mental illness, mental health conditions, etc.) are sometimes avoided, possibly to prevent labeling. In addition, certain programs do not directly specify their end-users’ mental health conditions. The use of this broad terminology seems purposeful and designed to avoid labeling and stigma to encourage greater participation. Moreover, most programs allow self-referral and do not demand a diagnosis, as well as offering community support rather than mental health treatment. This inclusive strategy is aimed to provide mental health services to any adolescent in distress, regardless of diagnosis. Finally, although all the program websites were checked several times throughout the search process, websites are dynamic and may be under construction or no longer exist.

## Conclusions and future directions

We reviewed 27 community-based programs for adolescents based on extensive literature and web search. More community-based services designed to address the developmental tasks and psychosocial needs of adolescents for social interaction are needed. Services that focus on the social aspects of personal recovery can facilitate crucial socialization tasks in adolescence, as well as the construction of a functioning identity, positive self-perception, and a sense of meaning (56). This is consistent with recent studies showing that prosocial behavior during adolescence has a positive effect on their mental health (57, 58). Furthermore, emphasizing social and leisure activities can enhance adolescent engagement in the programs and address the treatment gap, and therefore should be given more consideration in services for adolescents with MHC.

With the expected increase in MHC among adolescents, more psychosocial programs with an emphasis on social and leisure activities are needed to provide an adequate response to the rising need for the promotion of mental health among adolescents. Empirical data such as user and family satisfaction, specific needs and interests should be collected at the beginning, middle and end of participation in the programs to monitor participants’ acceptability and change. These practical implications should also be examined in future studies, such as by conducting focus groups of adolescents with MHC to learn which programs are more appealing to them, while considering their online visibility and advertising strategies. Data from the service team and mental health professionals who provide referrals to programs should also be gathered to better understand needs and enhance the service. This would help improve the expansion and implementation of programs and services and respond to users’ and their families’ needs. Thus, mental health professionals in the community are strongly encouraged to explore the programs available in their areas and advocate for community-based adolescent-oriented programs.

## Data availability statement

The original contributions presented in the study are included in the article, further inquiries can be directed to the corresponding author.

## Author contributions

HT: study design, data collection and analysis, and writing the manuscript. HO: study design, writing the manuscript, and supervision. All authors contributed to the article and approved the submitted version.
